# Optimizing PVA/Chitosan Films with Acid-Functionalized MWCNTs: A Multifaceted Study on Performance Enhancement

**DOI:** 10.3390/polym18080980

**Published:** 2026-04-17

**Authors:** Mukaddes Karataş, Buket Erzen, Şermin Deniz, Ercan Aydoğmuş, Ramazan Orhan

**Affiliations:** Faculty of Engineering, Department of Chemical Engineering, Fırat University, 23200 Elazığ, Türkiyerorhan@firat.edu.tr (R.O.)

**Keywords:** PVA/chitosan films, acid-functionalized MWCNT, nanocomposite films, mechanical properties, swelling behavior

## Abstract

Poly(vinyl alcohol)/chitosan (PVA/CS) biodegradable films reinforced with acid-functionalized multi-walled carbon nanotubes (f-MWCNTs) were fabricated via solution casting to investigate the effects of nanotube incorporation on structural, mechanical, thermal, dielectric, and physicochemical properties. Unlike conventional CNT-reinforced systems, this study focuses on the role of acid functionalization in improving nanotube dispersion and interfacial interactions, enabling simultaneous enhancement of multiple performance characteristics. Fourier transform infrared spectroscopy (FTIR) analysis confirmed strong intermolecular interactions between PVA/CS functional groups and carboxyl groups on f-MWCNTs, while scanning electron microscopy (SEM) revealed homogeneous nanotube dispersion at low loadings and partial aggregation at higher contents. X-ray diffraction (XRD) indicated that crystallinity was modified in a non-monotonic manner with increasing nanotube concentration due to competing nucleation and chain-restriction effects, while dielectric measurements showed an increase in dielectric constant from 3.78 to 4.27 as a result of enhanced interfacial polarization. The thermal conductivity improved from 0.195 to 0.247 W·m^−1^·K^−1^, and tensile strength increased from 19.8 to 24.5 MPa at 0.2 wt.% f-MWCNT, with elongation at break decreasing from 37.9% to 25.1%, reflecting increased stiffness. The degree of swelling and water solubility decreased with higher nanotube content, indicating reduced hydrophilicity and enhanced structural compactness. The results provide new insights into how surface-functionalized nanofillers can be used to tailor the multifunctional performance of biodegradable polymer nanocomposite films, highlighting their potential in advanced applications such as sustainable packaging, flexible electronics, sensors, and membrane technologies.

## 1. Introduction

The development of advanced multifunctional polymeric composites has become a promising area in the ever-evolving field of materials science, aimed at meeting the increasing demands for sustainability, improved performance, and environmental compatibility. The blend of polyvinyl alcohol (PVA) and chitosan (CS) is notable among the numerous polymeric systems studied due to its synergistic combination of mechanical strength, film-forming ability, and biodegradability [1,2]. PVA is a synthetic polymer that is widely discussed for its film-forming ability, emulsifying properties, and adhesive characteristics, which underpin its use in coatings, films, and wound dressings [3,4]. The hydrophilic nature of PVA and its hydrolyzed structure enable water compatibility and swelling behavior critical to emulsification and film formation, reinforcing its suitability for hydrophilic applications [5]. CS is derived from chitin through deacetylation, producing a polymer rich in amino and hydroxyl groups that enable extensive chemical modification and crosslinking [6,7]. It exhibits well-established antimicrobial activity, primarily due to electrostatic interactions between its positively charged backbone and microbial cell membranes; this activity depends on factors such as molecular weight, degree of deacetylation, and pH [8]. CS is also biodegradable through enzymatic (e.g., lysozyme-mediated) and hydrolytic mechanisms, supporting its use in biomedical and environmental applications. Its reactive functional groups allow diverse functionalization strategies (e.g., substitution, grafting, conjugation), enabling tailored solubility, charge, and bioactivity for specific uses [9].

The limitations of the PVA/CS films can be chiefly attributed to their inherent hydrophilic nature and structural properties, which impact their performance in various applications. The moisture sensitivity of the films leads to significant swelling and dimensional instability when exposed to humid environments, compromising their mechanical integrity over time [10,11]. Hydrophilic materials such as PVA and CS absorb moisture, decreasing the tensile strength as the absorbed water acts as a plasticizer. This diminishes the overall mechanical properties of the films, limiting their applicability in environments where moisture is prevalent [12]. Furthermore, the amorphous structure of PVA/CS blends, marked by weak intermolecular bonding, leads to suboptimal mechanical strength and barrier performance. These films lack the dense packing and crystalline properties that are usually necessary for resisting mechanical stress and effectively blocking moisture and gas permeation [13,14]. Thermal performance is another critical limitation faced by PVA/CS films. These materials exhibit low thermal resistance, which restricts their applications at elevated temperatures. Under heat, the polymer chains may degrade or lose structural stability, further undermining their operational efficacy in environments that may exceed moderate temperature thresholds. The entropic contributions from the polymeric chains, which should ideally enhance thermal stability, often result in poorer performance due to the hydrophilic character of the polymers involved [15,16]. These limitations collectively restrict the practical applicability of PVA/CS films in demanding fields such as food packaging, biomedical devices, and environmental membranes. Consequently, considerable research efforts have been directed toward improving their performance through the incorporation of nanofillers, particularly carbon-based nanomaterials, which exhibit unique physicochemical properties at the nanoscale [17,18,19].

In this regard, the integration of appropriate nanofillers presents an effective strategy to overcome the inherent shortcomings of PVA/CS systems. Carbon nanomaterials, especially multi-walled carbon nanotubes (MWCNTs), possess characteristics that directly address these limitations. Their high thermal conductivity can enhance heat dissipation and thermal stability, while their exceptional mechanical strength contributes to improved structural integrity and load-bearing capacity [20]. Although pristine MWCNTs are intrinsically hydrophobic, surface modification through acid functionalization introduces oxygen-containing functional groups (–COOH and –OH), which improve their dispersion within hydrophilic polymer matrices such as PVA and chitosan. This improved dispersion enables stronger interfacial interactions, which in turn enhance interfacial adhesion and promote the formation of a more compact polymer network. As a consequence, polymer chain mobility is restricted, and the available free volume within the matrix is reduced, thereby limiting the penetration and diffusion of water molecules. Moreover, the well-dispersed, high-aspect-ratio CNTs act as physical barriers that disrupt continuous diffusion pathways, forcing permeating species to follow more tortuous routes through the matrix. Together, these effects directly contribute to reduced moisture sensitivity and improved barrier performance [21].

Numerous studies have demonstrated that even at low loading levels, MWCNTs can significantly enhance the mechanical and functional properties of polymer matrices [22,23,24]. For instance, Aydın (2020) [25] explored the effects of integrating MWCNTs into PVA/CS films. The study concluded that incorporating MWCNTs improved thermal stability, as indicated by increased residual mass, thus supporting the idea that such modifications enhance the mechanical performance and stability of the films in varied environments [25]. Similarly, Dadfar et al. (2013) [26] investigated PVA thin films reinforced with MWCNTs, and their results highlighted that strong interfacial interactions between the MWCNTs and the PVA matrix result in noteworthy improvements in tensile strength, thereby reinforcing the potential of MWCNTs to enhance the performance of PVA composites for various applications. Despite these advances, a recurring challenge in MWCNT/polymer composites is the poor dispersion and weak interfacial adhesion between the hydrophobic nanotubes and hydrophilic polymers, which often leads to agglomeration and suboptimal reinforcement effects [27]. Functionalization using acid treatments can introduce hydrophilic carboxyl (–COOH) and hydroxyl (–OH) groups onto the MWCNTs, thus improving their compatibility and dispersion within the polymer matrix. Acid-functionalized MWCNTs (f-MWCNTs) exhibit enhanced interaction with the hydroxyl groups of PVA and the amino groups of CS, enabling better stress transfer during mechanical loading, which directly translates into improvements in the structural integrity of the composite [28]. Alexander et al. (2021) [29] demonstrated that functionalized carbon nanotubes (f-CNTs) disperse more effectively in PVA than pristine MWCNTs, with enhanced interfacial interactions observed via Fourier transform infrared spectroscopy (FTIR) and X-ray diffraction (XRD), leading to improved stress transfer in PVA-based composites. Studies have demonstrated that multiple processing parameters, including the concentration of f-MWCNTs, the level of functionalization, and the film fabrication method, play a critical role in determining the mechanical and thermal properties of CNT/polymer nanocomposites [30,31]. Such insights are essential not only for optimizing composite formulations but also for expanding their applicability in areas such as packaging, biomedical, and environmental systems, where performance reliability and sustainability are paramount [32].

Although the literature includes various studies on the integration of MWCNTs into PVA/CS-based films, comprehensive investigations focusing on how acid-functionalization alters the dispersion, interfacial bonding, and resulting performance characteristics of these composites are still quite limited. Currently, no thorough investigation has been conducted to systematically examine the structure–property connections in PVA/CS films strengthened with f-MWCNTs across a variety of filler loadings, nor one that assesses several performance criteria such as mechanical strength, thermal properties, swelling and solubility in water, and morphology. This gap in the literature underscores the need for an integrated, experimental approach to understand and optimize these nanocomposites for real-world applications. The present study aims to address this need by investigating the effects of incorporating varying concentrations of f-MWCNTs into PVA/CS composite films, with a focus on optimizing their mechanical, thermal, and structural properties. By doing so, this study not only advances the design of high-performance biodegradable films but also lays a foundation for future research in areas such as active food packaging, biomedical membranes, and environmentally responsive materials, where tailored nanocomposite properties are essential for functionality and regulatory compliance.

## 2. Materials and Methods

### 2.1. Materials

PVA (average molecular weight ~89,000–98,000 g·mol^−1^, 99+% hydrolyzed) was purchased from Sigma-Aldrich (Darmstadt, Germany). CS (medium molecular weight, degree of deacetylation ~75–85%) was obtained from BLDpharm (Shanghai, China). Pristine MWCNTs (outside diameter: 8–18 nm, purity > 96%) were supplied by Nanografi (Ankara, Türkiye). Sulfuric acid (H_2_SO_4_, 98%) and nitric acid (HNO_3_, 70%) were purchased from Merck (Darmstadt, Germany) and used for the functionalization of MWCNTs. Acetic acid (CH_3_COOH, glacial) and distilled water were used throughout the experiments. All chemicals were of analytical grade and used without further purification.

### 2.2. Acid Functionalization of MWCNTs

The acid functionalization procedure for multi-walled carbon nanotubes (MWCNTs) was executed utilizing a modified reflux technique [33]. Initially, 1 g of pristine MWCNTs was introduced into a round-bottom flask containing a 3:1 (*v*/*v*) mixture of concentrated sulfuric acid and nitric acid. The mixture was then subjected to sonication for 30 min to achieve effective dispersion of the nanotubes. Following this step, the flask was heated to 80 °C and refluxed for 4 h while maintaining continuous stirring. Upon cooling to room temperature, the resulting suspension was diluted with 100 mL of distilled water and filtered through a 0.45 µm PTFE membrane to collect the solid residue. This solid was then thoroughly washed with distilled water multiple times until the washings reached a neutral pH. Finally, the acid-functionalized MWCNTs (denoted as f-MWCNTs) were dried in a vacuum oven at a temperature of 60 °C for 24 h and subsequently stored in a desiccator for later use.

### 2.3. Preparation of Composite Films

The preparation of PVA/CS composite films reinforced with f-MWCNTs involved a systematic approach to ensure uniformity and optimal properties. Initially, a 8% (*w*/*v*) PVA solution was prepared by dissolving PVA in distilled water at 85 °C, with constant stirring until a clear and homogeneous solution was achieved. Concurrently, a 2% (*w*/*v*) CS solution was prepared in a 1% (*v*/*v*) aqueous acetic acid, stirred at room temperature for 12 h until fully dissolved. Equal volumes of the PVA and chitosan solutions were then combined to create the PVA/CS blend, which was stirred for an additional two hours to ensure complete mixing. The incorporation of f-MWCNTs into the blend was carried out by adding the nanotubes in varying weight percentages (0.2, 0.5, 0.8, and 1.0 wt.%) relative to the total polymer content. The f-MWCNTs were initially dispersed in distilled water using ultrasonication for 30 min before being added gradually to the PVA/CS mixture under intense stirring conditions. The combined mixture then underwent a further 15 min of sonication to remove any remaining agglomerates and ensure a uniform dispersion of the nanotubes. Following this, the composite solution was poured into clean glass Petri dishes and left to dry at room temperature for 48 h in a dust-free environment. After drying, the films were carefully peeled off and conditioned in a desiccator at 50% relative humidity (RH) and 23 °C for a minimum of 48 h before undergoing characterization.

### 2.4. Characterization of Composite Films

In this study, FTIR was performed using a Shimadzu IRSpirit QATR-S spectrometer (Kyoto, Japan), which is designed to execute high-precision spectral measurements and offers advanced capabilities for material characterization. The purpose of the FTIR analysis was twofold: firstly, to confirm successful acid functionalization of the MWCNTs, and secondly, to assess molecular interactions within the PVA/CS/f-MWCNT composite films. The FTIR spectra were obtained over a wavenumber range of 400–4000 cm^−1^, covering a broad spectrum of vibrational modes.

Scanning electron microscopy (SEM) analysis was carried out using a Zeiss EVO MA 10 scanning electron microscope (Oberkochen, Germany) to obtain high-resolution images of the sample surface morphology. The primary objective of using SEM in this context was to examine the dispersion of f-MWCNTs within the polymer matrix of the composite films. Films were sputter-coated with a thin layer of gold and imaged at an accelerating voltage of 10–15 kV.

The crystalline structure and phase behavior of the PVA/chitosan/f-MWCNT composite films were analyzed using XRD. The measurements were carried out on a Rigaku MiniFlex II diffractometer (Tokyo, Japan) equipped with Cu Kα radiation (λ = 1.5406 Å), operated at 40 kV and 15 mA. Scans were performed over a 2θ range of 5° to 60° at a scanning rate of 6°/min with a step size of 0.01°. Film samples were cut into small rectangular pieces and mounted flat on the sample holder without any special treatment. The obtained diffraction patterns were used to evaluate the crystallinity index and to investigate the effect of f-MWCNT incorporation on the molecular ordering of the PVA/CS matrix. The interplanar spacing values were obtained from the peak positions using Bragg’s law, while crystallite sizes were estimated from the fitted diffraction peaks using the Scherrer equation. These values were extracted from the XRD peak-fitting output provided by the analysis software.

The tensile properties of composite films were tested using a universal testing machine (Shimadzu Autograph AGS-X, Kyoto, Japan) as specified by the ASTM D882 standards [34,35]. Uniform strips were cut and tested at a crosshead speed of 1 mm/min. The tensile strength (TS) was calculated as the maximum load at the break divided by the original cross-sectional area of the specimen, and the elongation at break (EAB) was given as the percentage increase in length before the fracture occurred. Five individual specimens were used to test each composition, with the average values being reported to guarantee data reliability.

The dielectric properties of the composite films were evaluated using a Fytronix precision LCR meter (Elazığ, Türkiye) operating over the frequency range of 1 kHz to 1 MHz, in accordance with ASTM D150 standards [36]. Circular film samples with a diameter of 20 mm were cut and mounted between two parallel plate electrodes in a capacitor configuration. Measurements were performed at 25 ± 1 °C under ambient laboratory conditions. For each formulation, at least three separate measurements were taken, and the mean dielectric constant was calculated to ensure reliability.

Thermal conductivity measurements of the composite films were performed using a steady-state heat flow thermal conductivity analyzer (TLS-100, Thermtest Inc., Fredericton, NB, Canada). Rectangular specimens were placed between a controlled-temperature heat source and a cooled sink, ensuring uniform contact at both interfaces. The steady heat transfer rate through each sample was recorded, and the thermal conductivity (*k*) was determined based on the measured temperature gradient and sample dimensions. Tests were conducted in triplicate for each formulation to ensure reproducibility, and the results were subjected to statistical evaluation to examine the effect of filler content on the thermal performance of the films.

The swelling behavior of the films was determined by immersing 2 × 2 cm pre-weighed dry samples in distilled water at 25 ± 1 °C [37]. At predetermined time intervals (1, 2, 4, 8, 12, and 24 h), the samples were removed, gently blotted with filter paper to remove excess surface water, and weighed using an analytical precision balance (Mettler Toledo MA104E, Greifensee, Switzerland). After weighing, samples were returned to the same container for continued immersion until the next time point. The swelling equilibrium was deemed to be achieved when the weight variation between two successive measurements was less than 1%. All tests were conducted in triplicate, and the average swelling values were reported for each immersion time. The swelling ratio (SR) at each interval was calculated as:

SR (%) = (W_t_ − W_d_)/W_d_ × 100, (1)

where W_d_ is the initial weight and W_t_ is the swollen weight.

Water solubility was determined by immersing pre-weighed dry film samples (W_i_) in 50 mL of distilled water at 25 ± 1 °C for 24 h. After immersion, the remaining insoluble portion was collected, dried in an oven at 50 °C until constant weight (W_f_), and weighed. All tests were carried out in triplicate, and the mean values were used for analysis. The solubility percentage (S) was calculated as:

S (%) = (W_i_ − W_f_)/W_i_ × 100, (2)

where W_i_ is the initial weight and W_t_ is the final dry weight.

## 3. Results

### 3.1. FTIR Analysis

**Figure 1:**
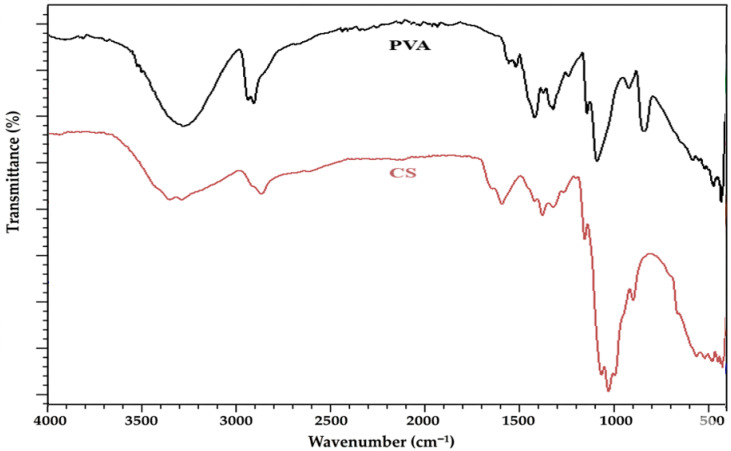
FTIR spectra of pure PVA and CS.

Figure 1 presents the FTIR spectra of pure PVA and CS, highlighting their characteristic functional groups. The PVA spectrum exhibits a broad absorption band around 3200–3400 cm^−1^ corresponding to O–H stretching vibrations, along with peaks near 2940 cm^−1^ attributed to C–H stretching. The bands observed at approximately 1730 cm^−1^ and 1090 cm^−1^ are associated with residual acetate groups and C–O stretching vibrations, respectively. In contrast, the CS spectrum shows a broad O–H/N–H stretching band in the same region (3200–3400 cm^−1^), along with characteristic amide bands around 1650 cm^−1^ (amide I) and 1550 cm^−1^ (amide II), confirming the presence of amino functional groups. The overlapping hydroxyl and amine bands in both spectra indicate the strong potential for hydrogen bonding interactions between PVA and CS, which play a key role in the formation and compatibility of the blended nanocomposite film system. These observations establish a reference for evaluating the spectral modifications in the PVA/CS blend and its nanocomposites.

**Figure 2:**
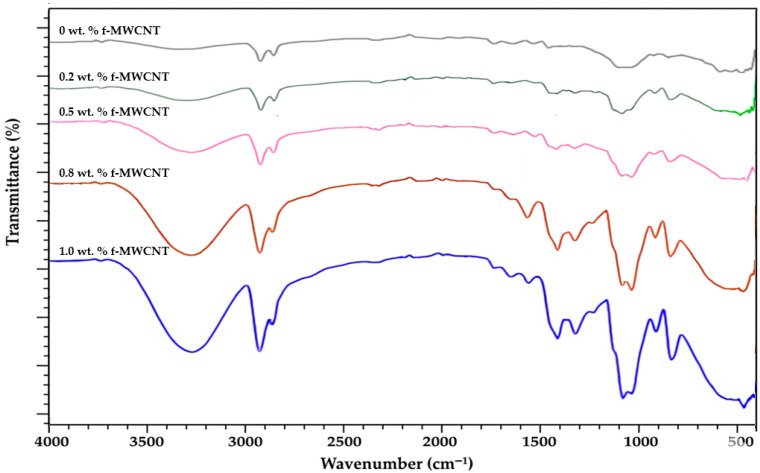
FTIR spectra of PVA/CS (80/20 wt.%) films containing different concentrations (0–1 wt.%) of f-MWCNTs.

Figure 2 shows the FTIR spectra of neat PVA/CS films and PVA/CS composite films reinforced with various f-MWCNT concentrations. The neat PVA/CS (80/20 wt.%) nanocomposite film exhibited characteristic absorption bands associated with its functional groups: a broad band at ~3300–3400 cm^−1^ corresponding to O–H and N–H stretching vibrations, peaks near 2930 cm^−1^ assigned to C–H stretching, and bands at ~1640 cm^−1^ and ~1550 cm^−1^ related to amide I (C=O stretching) and amide II (N–H bending) of CS, respectively [38]. The absorption peak around 1080–1150 cm^−1^ is attributed to the C–O–C stretching vibrations of the PVA backbone and the C–O stretching of the polysaccharide structure of chitosan. Additionally, the band observed near 1420–1450 cm^−1^ corresponds to CH_2_ bending vibrations, while the peak around 1320–1370 cm^−1^ is associated with C–N stretching of the amine groups present in chitosan. The characteristic peak near 890–920 cm^−1^ is related to the saccharide structure of chitosan, confirming the presence of glycosidic linkages. These functional group vibrations verify the successful blending of PVA and chitosan and indicate the formation of intermolecular hydrogen bonding interactions between hydroxyl and amino groups, contributing to the structural integrity of the polymer matrix [39].

Several significant changes in the FTIR spectra were observed with the incorporation of f-MWCNTs, indicating intermolecular interactions between the polymer matrix and the nanofiller. The broad O–H/N–H stretching band of neat PVA/CS, initially centered at approximately 3340 cm^−1^, became wider and shifted slightly toward lower wavenumbers (3325–3315 cm^−1^) with increasing f-MWCNT content. This broadening and shift are consistent with enhanced hydrogen bonding interactions between polymer matrix functional groups (–OH, –NH_2_) and the carboxyl groups introduced on the f-MWCNT surface during acid functionalization. The observed downshift may indicate an increase in hydrogen-bonding strength and enhanced interfacial interactions between the polymer matrix and f-MWCNTs. However, it should be noted that this spectral region contains overlapping contributions from O–H and N–H vibrations; therefore, these interpretations are based on qualitative observations rather than detailed deconvolution analysis [40,41].

Moreover, the characteristic amide I band observed near 1642 cm^−1^ and the amide II band around 1555 cm^−1^ showed a gradual decrease in intensity and slight peak shifts (to approximately 1635 cm^−1^ and 1548 cm^−1^, respectively) as the f-MWCNT concentration increased. The observed attenuation and downshifts of amide I and II bands reflect restricted molecular mobility and altered vibration environments of CS–PVA segments due to nanotube–polymer interfacial interactions. Such changes are consistent with carbon nanotube (CNT)-functional group interactions (e.g., carboxyl, amine, hydroxyl on f-MWCNTs) engaging with CS/PVA functional groups, effectively perturbing the local vibrational landscape of amide linkages. The downshift indicates strengthening or modification of hydrogen-bonding networks and/or covalent-like interfacial contacts that constrain amide group motions. Khoerunnisa et al. (2018) document FTIR shifts in CS/PVA/MWCNT systems, noting amide-related region changes upon CNT incorporation and linking them to CNT–polymer interfacial interactions that affect chain mobility [42].

Changes were also observed in the 1085–1145 cm^−1^ region associated with C–O–C stretching vibrations, where a minor shift toward 1090–1100 cm^−1^ suggests alterations in the hydrogen bonding network of the PVA/chitosan matrix. The shift to slightly higher or lower wavenumbers, depending on CNT functionalization and dispersion, is a recognized signature of altered hydrogen-bond strength and interfacial coupling in PVA-containing CNT composites. In this context, Zohora et al. (2019) observed changes in the C–O–C region and perturbations in the O–H region of PVA/CS/MWCNT systems, consistent with strengthened interfacial hydrogen bonding and modifications of the C–O environment [43]. Additionally, weak absorption bands appearing in the 620–780 cm^−1^ region became more noticeable at higher MWCNT loadings, which can be attributed to skeletal vibration modes of the carbon nanotube structure. This spectral range includes various CNT skeletal vibration modes, such as C–C bending and out-of-plane motions, as well as other low-frequency CNT-related features. Increasing CNT content intensifies these signatures, indicating effective filler incorporation and the formation of CNT network vibrations within the composite. These observations are consistent with CNT-specific spectral fingerprints previously reported in PVA/CS systems and other CNT-reinforced biopolymer matrices [25,29].

Overall, the FTIR analysis confirms the successful incorporation of f-MWCNTs into the PVA/CS matrix and highlights the development of strong interfacial interactions. These spectral features indicate improved molecular compatibility between components, which underpins the observed enhancements in mechanical strength, thermal stability, and barrier performance of the PVA/CS/MWCNT composite films.

### 3.2. SEM Analysis

**Figure 3:**
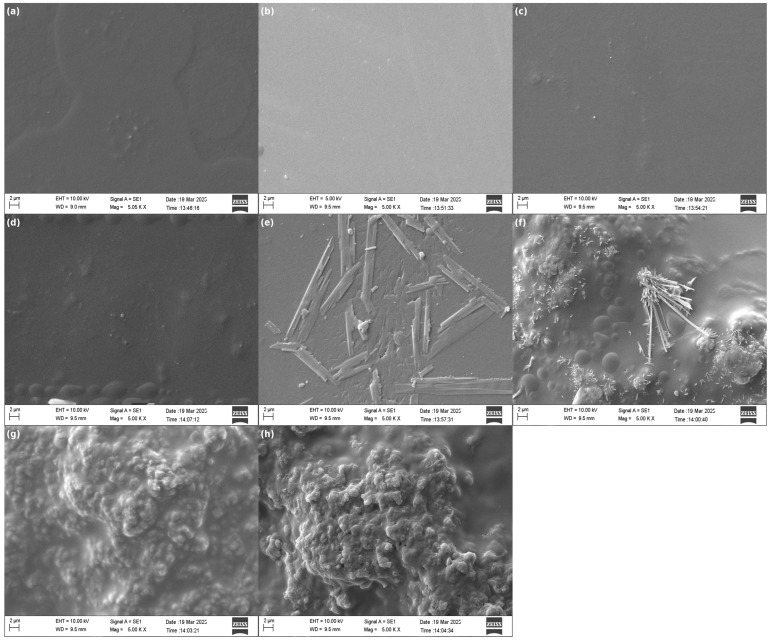
SEM micrographs of the studied samples: (**a**) pure PVA (10 wt.%); (**b**) pure chitosan (CS, 3 wt.%); (**c**) PVA/CS blend; PVA/CS composites with f-MWCNT at (**d**) 0.2 wt.%, (**e**) 0.5 wt.%, (**f**) 0.8 wt.%, and (**g**) 1.0 wt.%; and (**h**) PVA/CS composite with unfunctionalized MWCNT (1.0 wt.%).

The surface morphologies of the pure polymers, their blend, and the f-MWCNT reinforced PVA/CS nanocomposite films were examined using SEM, as shown in Figure 3. The SEM micrographs of pure PVA (10 wt.%) (Figure 3a) revealed a smooth and homogeneous surface, indicative of the uniform polymeric matrix without any phase separation. This observation aligns with multiple reports where PVA films exhibit smooth surfaces and continuous morphology in SEM, reflecting uniform chain distribution and limited phase separation under suitable casting conditions [44]. Similarly, pure chitosan (3 wt.%) (Figure 3b) displayed a continuous, compact structure, consistent with its semicrystalline nature. The reported compact, continuous morphology for CS aligns with typical SEM observations of CS films that lack significant phase separation and exhibit coherent, continuous surfaces at modest thicknesses [45].

The PVA/CS blend (Figure 3c) showed a slightly rougher morphology compared to the individual polymers, suggesting intermolecular interactions between PVA and CS, which promote partial miscibility in the blend. The observation of a slightly rougher morphology for PVA/CS blends compared with the pure components is in line with literature reporting intermolecular hydrogen bonding and partial miscibility contributing to subtle surface texturing rather than complete phase separation [46,47]. Upon the addition of 0.2 wt.% f-MWCNTs (Figure 3d), the surface of the PVA/CS composite exhibited minor roughness with a few dispersed nanotubes visible. This indicates successful incorporation of f-MWCNTs without significant agglomeration. Studies of CS/PVA/CNT systems show that functionalization (e.g., carboxylation, amine groups) facilitates hydrogen bonding and interfacial interactions that promote better dispersion and minimize visible agglomerates in SEM at low CNT contents [48]. The nanocomposite surface became noticeably rougher at a 0.5 wt.% f-MWCNT loading (Figure 3e), and the presence of rod-like f-MWCNT structures suggests improved interfacial adhesion, which can facilitate stress transfer under mechanical loading. Further increasing the filler content to 0.8 wt.% and 1.0 wt.% (Figure 3f,g) resulted in denser and more irregular surfaces, with visible clusters of f-MWCNTs. This morphology indicates partial aggregation at higher loadings, which may adversely affect homogeneity but can still contribute to mechanical reinforcement if well dispersed locally. Notably, the unfunctionalized MWCNT at 1.0 wt.% (Figure 3h) demonstrated a highly aggregated surface, emphasizing the importance of surface functionalization to improve compatibility with the polymer matrix. The absence of surface functional groups reduces interfacial bonding with CS/PVA, increasing propensity for CNT–CNT agglomeration, particularly at higher loadings; this leads to coarse, aggregated surfaces in SEM and poor dispersion quality [49].

SEM analysis confirms that functionalization of MWCNTs enhances dispersion within the PVA/CS matrix, leading to a more uniform morphology at lower to moderate loadings (0.2–0.8 wt.%), whereas higher filler concentrations tend to promote aggregation. These morphological observations correlate with expected mechanical and thermal properties, as well-dispersed nanotubes contribute to improved stress transfer and thermal stability, whereas agglomerates can act as defect sites.

### 3.3. Structural Analysis by XRD

**Figure 4:**
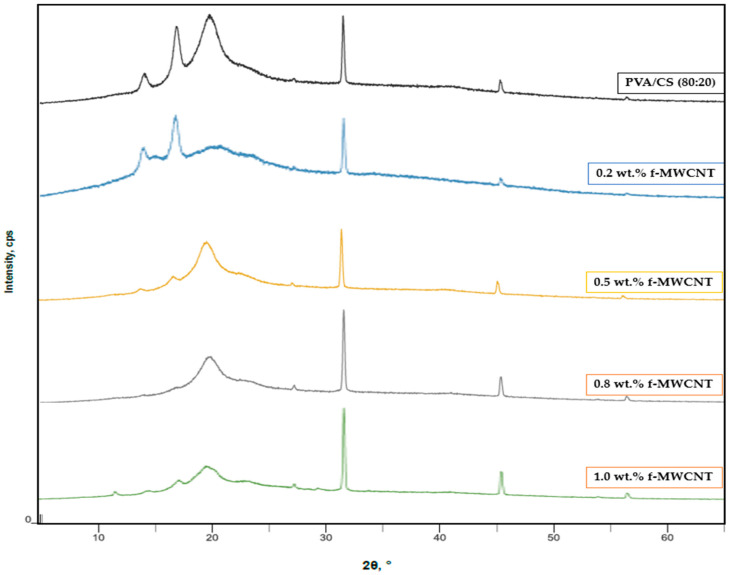
XRD patterns of PVA/CS composite films and PVA/CS- f-MWCNT nanocomposites with varying filler loadings: PVA/CS (top), PVA/CS/f-MWCNT (0.2 wt.%), PVA/CS/f-MWCNT (0.5 wt.%), PVA/CS/f-MWCNT (0.8 wt.%) and PVA/CS/f-MWCNT (1.0 wt.%).

The XRD patterns of PVA/CS and PVA/CS reinforced with different concentrations of functionalized MWCNTs (0–1 wt.%) are shown in Figure 4. The neat PVA/CS blend exhibits a broad diffraction peak centered at 2θ ≈ 19–20°, corresponding to the (101) crystalline plane and characteristic of the semicrystalline structure of PVA. XRD analyses of PVA-rich matrices consistently show a broad peak in this region, attributed to semicrystalline PVA domains formed via interchain hydrogen bonding [50]. This peak results from intermolecular hydrogen bonding interactions between the hydroxyl groups of PVA and the amino and hydroxyl functional groups of chitosan, confirming good compatibility between the two polymers. Ramesan et al.’s (2024) reviews on PVA-based blends (including CS-containing systems) consistently connect hydrogen-bonding at the PVA–CS interface with changes in crystallinity observable by XRD and supported by FTIR band perturbations [51].

The crystallinity index (CI) was calculated using a quantitative area-based method, which was derived from the results of peak fitting, as shown below:

CI (%) = (A_c_/(A_c_ + A_a_)) × 100 (3)

where A_c_ and A_a_ represent the crystalline and amorphous peak areas obtained from XRD profile fitting. The calculated CI values were 71.97%, 78.96%, 88.05%, 89.17%, and 83.53% for films containing 0, 0.2, 0.5, 0.8, and 1.0 wt.% f-MWCNT, respectively, while the film containing 1.0 wt.% unfunctionalized MWCNT exhibited a CI of 78.59%. These values provide a quantitative basis for understanding the structural modifications induced by nanotube incorporation.

In addition to crystallinity analysis, structural parameters including interplanar spacing (d-spacing) and crystallite size were obtained from the fitted XRD peaks. The diffraction peaks for all samples remained within a narrow range (2θ ≈ 19.8–20.3°), indicating that the fundamental crystalline structure of the PVA/CS matrix was preserved upon nanotube incorporation. However, subtle variations in peak position and width were observed, reflecting changes in local chain packing and crystalline domain size.

The d-spacing values showed a slight decrease from 4.48 Å for the neat film to approximately 4.37 Å at 0.8 wt.% f-MWCNT, suggesting closer chain packing due to enhanced intermolecular interactions. At 1.0 wt.% f-MWCNT, the d-spacing increased slightly to 4.41 Å, indicating a minor relaxation of the polymer structure, likely due to increased nanotube interactions and partial aggregation. A similar trend was observed for the crystallite size, which increased from 3.84 nm for the neat film to a maximum of 6.46 nm at 0.8 wt.% f-MWCNT, followed by a slight decrease at higher loading. This behavior is consistent with the SEM observations, where improved dispersion at moderate filler content is followed by increased aggregation at higher concentrations, which can hinder the development of well-ordered crystalline domains.

These results reveal a non-monotonic evolution of crystallinity and structural parameters, indicating that f-MWCNTs play a dual role in the PVA/CS matrix. At low to moderate concentrations, functionalized nanotubes act as effective nucleating agents, promoting polymer chain alignment and crystallite growth through strong interfacial interactions. This is reflected in the increase in both crystallinity index and crystallite size. At higher loadings (1.0 wt.%), increased nanotube–nanotube interactions and partial aggregation begin to restrict polymer chain mobility and reduce the efficiency of crystal formation, leading to a slight decrease in crystallinity [52,53].

**Figure 5:**
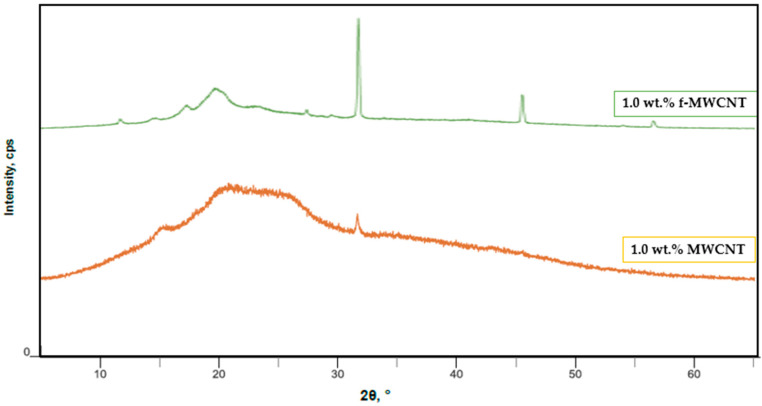
Comparison of XRD patterns of PVA/CS composites reinforced with 1 wt.% f-MWCNT and 1 wt.% MWCNT.

Figure 5 presents a comparison between nanocomposite films containing 1 wt.% f-MWCNT and 1 wt.% unfunctionalized MWCNT. The f-MWCNT-reinforced nanocomposite film exhibits a relatively sharper and more defined diffraction peak at 2θ ≈ 19–20°, indicating improved structural organization and more effective interfacial compatibility with the PVA/CS matrix. In contrast, the nanocomposite film containing unfunctionalized MWCNT shows broader peaks with lower intensity, reflecting poorer dispersion and increased aggregation. The absence of surface functional groups in pristine nanotubes limits their interaction with the hydrophilic polymer matrix, leading to CNT-rich regions that disrupt uniform chain packing and reduce crystallization efficiency [29,54].

In comparison, the film containing 1.0 wt.% unfunctionalized MWCNT exhibited lower crystallinity and smaller crystallite size than the corresponding functionalized system, highlighting the importance of surface functionalization in improving dispersion and interfacial compatibility. Functionalized nanotubes provide a more effective nucleation environment, whereas poor dispersion in unfunctionalized systems limits structural ordering. Overall, the XRD results demonstrate that acid-functionalized MWCNTs effectively tailor the structural organization of PVA/CS nanocomposites by enhancing crystallinity and promoting the formation of larger and more ordered crystalline domains at optimal filler concentrations.

### 3.4. Characterization of Mechanical, Dielectric, and Thermal Properties

**Figure 6:**
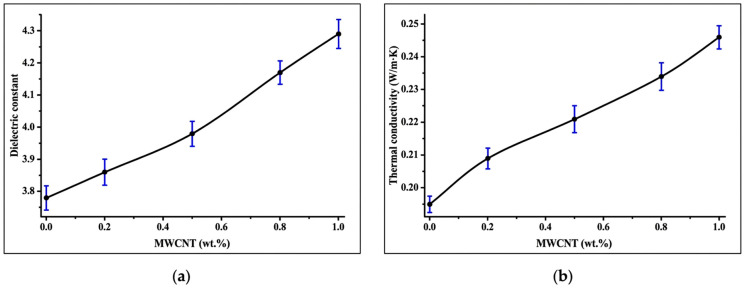
(**a**) Dielectric constant and (**b**) thermal conductivity of PVA/CS composite films as a function of f-MWCNT content (0–1 wt.%).

The dielectric constant of the PVA/CS composite films increased gradually with increasing content of acid-functionalized MWCNTs, as presented in Figure 6a. The neat PVA/CS film exhibited a dielectric constant of 3.78, which can be attributed to the intrinsic dipolar polarization arising from hydroxyl (–OH) and amino (–NH_2_) groups present in the polymer structure. The addition of 0.2 wt.% f-MWCNT resulted in a slight increase in dielectric constant to 3.85, suggesting the influence of interfacial polarization effects due to the presence of conductive nanofillers within the insulating polymer matrix. Studies on CNT-filled polymer matrices, such as PVA- and chitosan-containing systems, have found that the dielectric constant increases with rising CNT content, which can be attributed to MWS interfacial polarization. Functionalized CNTs improve the interfacial contact between the conductive filler and dielectric matrix, enabling enhanced charge accumulation at interfaces under an applied field [55,56].

A further increase in f-MWCNT concentration led to a continuous improvement in dielectric performance, peaking at 4.27 at a 1 wt.% loading. The observed increase can be attributed to the Maxwell–Wagner–Sillars (MWS) interfacial polarization mechanism, in which charge accumulation occurs at the interfaces between the conductive f-MWCNTs and the dielectric polymer matrix. Acid functionalization introduces oxygen-containing functional groups such as –COOH onto the nanotube surface, enhancing dispersion and encouraging stronger interfacial interactions with PVA and chitosan chains. The process enhances dipole orientation and facilitates polarization in the presence of an applied electric field [57].

The gradual increase in dielectric constant with nanofiller concentration indicates enhanced polarization behavior and improved charge storage capability of the composite films. Notably, the absence of a pronounced increase in dielectric loss suggests that the system likely remains below the electrical percolation threshold over the studied filler range (0–1 wt.% f-MWCNT). This observation aligns with literature showing that near or below percolation, dielectric loss remains relatively modest because long-range conductive networks have not formed and conduction losses are limited; interfacial polarization remains the dominant mechanism governing dielectric response in this regime [58].

As seen in Figure 6b, the thermal conductivity of the PVA/CS composite films increased progressively with increasing concentrations of acid-functionalized MWCNTs. The neat PVA/CS film exhibited a thermal conductivity of 0.195 W·m^−1^·K^−1^, which is consistent with the typical low heat transfer capability of polymeric materials due to their amorphous structure and phonon scattering within the polymer chains. With the addition of 0.2 wt.% f-MWCNT, the thermal conductivity increased slightly to 0.208 W·m^−1^·K^−1^, indicating that even a small amount of nanotubes contributed to improved heat transfer pathways within the polymer matrix. A more noticeable increase was observed at 0.5 wt.% f-MWCNT, where the thermal conductivity reached 0.228 W·m^−1^·K^−1^. This enhancement can be attributed to the high intrinsic thermal conductivity of MWCNTs and their ability to facilitate phonon transport across the composite structure. Studies on CNT-filled polymer matrices have shown that thermal conductivity increases with CNT content due to the formation of more efficient phonon transport pathways within the polymer matrix. In such systems, functionalization improves CNT–polymer interfacial compatibility, reduces interfacial thermal resistance, and promotes more effective heat transfer across the filler–matrix interface, thereby enhancing the overall thermal transport performance of the composite [59,60].

Further increases in nanofiller content continued to enhance thermal transport performance, reaching 0.233 W·m^−1^·K^−1^ at 0.8 wt.% and 0.247 W·m^−1^·K^−1^ at 1 wt.% f-MWCNT, representing the highest thermal conductivity obtained in this work. The progressive improvement suggests the formation of increasingly interconnected heat-conducting networks within the composite as the nanotube concentration increased. Surface functionalization of MWCNTs likely promoted better compatibility with the PVA/CS matrix, minimizing interfacial thermal resistance and supporting more effective phonon propagation throughout the material. These results demonstrate that the incorporation of functionalized nanotubes provides a controlled and efficient strategy to improve the thermal transport capability of biodegradable polymer films without compromising their structural integrity.

**Figure 7:**
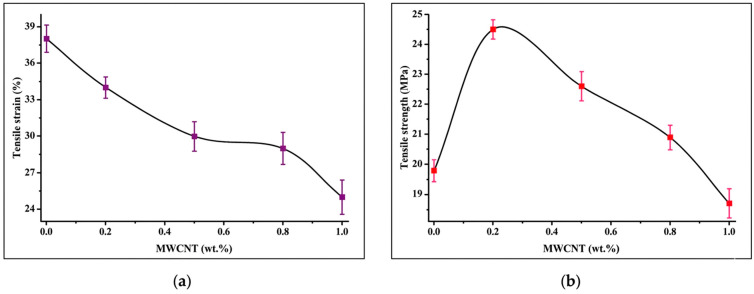
(**a**) Tensile strain and (**b**) tensile strength of PVA/CS composite films as a function of f-MWCNT content (0–1 wt.%).

The variation in elongation at break of PVA/CS composite films with increasing f- MWCNT content is illustrated in Figure 7a. The neat PVA/CS film exhibited the highest tensile strain value of 37.9%, reflecting the relatively high flexibility of the polymer blend due to the mobility of PVA and chitosan chains and the presence of intermolecular hydrogen bonding interactions, which allowed significant elongation before the addition of CNTs. The incorporation of 0.2 wt.% f-MWCNTs resulted in a reduction in tensile strain to 34.1%, a phenomenon primarily attributed to the restricted mobility of polymer chains within the matrix. The trend intensified at a 0.5 wt.% loading, where the elongation at break decreased to 30.2%, indicating an increase in composite stiffness. The progressive reduction in ductility is driven by increased interactions between the functionalized nanotubes and the hydroxyl and amino groups of the PVA/chitosan blend, specifically through hydrogen bonding. The robust interactions effectively immobilize the polymer segments by constraining their movement under tensile stress.

At higher filler concentrations of 0.8 wt.% and 1.0 wt.%, the tensile strain reached 29.4% and 25.1%, respectively. These findings highlight the significance of f-MWCNTs as strong reinforcing components that enable the creation of a stiff, interconnected framework. Surface functionalization enhances the nanocomposite’s structural compactness and interfacial adhesion, while also promoting superior dispersion and suppressing segmental mobility. Zohora et al. (2019) [43] found in their study that the mechanical properties of PVA/CS blends are altered by the addition of MWCNTs, with the extent of the change depending on the concentration of CNTs. In a 50:50 PVA/CS system, they found that elongation at break initially increases at low CNT loadings but decreases at higher loadings, possibly due to agglomeration and stress concentration, suggesting a CNT-dependent change in ductility [43].

These results demonstrate that the addition of f-MWCNTs gradually reduces the flexibility of PVA/CS films while improving structural rigidity. The controlled decrease in elongation at break suggests that the composite films maintain sufficient flexibility for practical use while benefiting from improved mechanical stability.

The tensile strength of the PVA/CS composite films was significantly influenced by the f-MWCNT content, as shown in Figure 7b. The neat PVA/CS film, containing 0 wt.% f-MWCNT, exhibited a tensile strength of 19.8 MPa. The incorporation of 0.2 wt.% f-MWCNT increased the tensile strength to 24.5 MPa, indicating improved load-bearing capacity of the composite structure. This enhancement can be attributed to the homogeneous dispersion of functionalized nanotubes within the polymer matrix, which promotes strong interfacial interactions such as hydrogen bonding between the hydroxyl groups of PVA, the amino groups of chitosan, and the oxygen-containing functional groups on the surface of f-MWCNTs. These interactions facilitate efficient stress transfer from the polymer matrix to the rigid nanotube framework, thereby improving the mechanical performance of the films.

Further increases in f-MWCNT loading beyond 0.2 wt.% resulted in a progressive decline in tensile strength values, with 22.6 MPa, 20.9 MPa, and 18.7 MPa recorded for 0.5, 0.8, and 1 wt.% f-MWCNT, respectively. The reduction in tensile strength at higher filler concentrations is likely associated with the increased tendency of nanotubes to form agglomerates due to strong van der Waals interactions between adjacent CNT structures. These agglomerates can act as structural defects and localized stress concentration regions, leading to premature crack initiation and propagation under tensile loading. In addition, excessive filler incorporation may restrict the mobility and uniform arrangement of polymer chains, thus limiting effective stress distribution throughout the composite matrix.

The observed trend suggests that a small amount of f-MWCNT improves load transfer efficiency and reinforces the polymer network, whereas higher loadings result in diminished tensile performance due to aggregation effects and disruption of matrix continuity. Similar behavior has been reported for CNT-reinforced PVA-based and chitosan/PVA blend systems, where mechanical enhancement is achieved at low nanotube contents but declines at higher concentrations due to filler–filler interactions dominating over polymer–filler interactions [61,62].

Overall, compared with other nanofiller methods reported in the literature, the PVA/CS films reinforced with f-MWCNTs presented in this study obtain a balanced and stepwise improved performance at relatively low filler loadings. Graphene- and GO-based composites often demonstrate higher dielectric or thermal responses due to their planar structure; however, their performance is highly dependent on dispersion quality and is frequently accompanied by aggregation-related limitations [63]. Similarly, ceramic-filled systems such as BaTiO_3_ and Al_2_O_3_ can provide significant improvements in dielectric and thermal properties, but typically require higher filler loadings and may compromise flexibility and processability. In contrast, the use of acid-functionalized MWCNTs enables simultaneous improvement in dielectric behavior, thermal transport, and mechanical strength at relatively low filler concentrations, while maintaining structural integrity and flexibility of the polymer matrix [64]. Collectively, these findings reinforce the conclusion that functionalized MWCNTs provide an effective strategy for achieving simultaneous improvements in dielectric response, thermal transport, and mechanical robustness while maintaining processability and flexibility—advantages particularly relevant for flexible electronics, capacitive energy storage, and insulating applications.

### 3.5. Swelling and Solubility Characteristics

The swelling behavior of PVA/CS composite films was significantly influenced by f-MWCNT content, as presented in Table 1. Both PVA and chitosan are highly hydrophilic polymers containing abundant hydroxyl and amino functional groups that readily form hydrogen bonds with water molecules, resulting in considerable swelling in aqueous environments. Accordingly, the neat PVA/CS film (0 wt.% f-MWCNT) exhibited the highest swelling degree of 140%, reflecting the high mobility of polymer chains and strong affinity for water.

With the incorporation of f-MWCNTs, the swelling degree gradually decreased from 140% to 118% as the filler content increased. This reduction can be attributed to enhanced intermolecular interactions between the functionalized nanotubes and the polymer matrix. The oxygen-containing functional groups present on the surface of f-MWCNTs promote hydrogen bonding with the hydroxyl groups of PVA and the amino groups of chitosan, leading to improved interfacial compatibility and formation of a more compact structure. As a result, polymer chain mobility becomes restricted and the free volume within the matrix decreases, limiting the diffusion of water molecules into the composite. Furthermore, the relatively uniform dispersion of nanotubes, particularly at lower concentrations, creates a tortuous diffusion pathway that hinders water penetration and contributes to reduced swelling capacity. Similar observations have been reported for CNT-reinforced PVA/CS and related polymer systems, where strong filler–polymer interactions suppress water uptake and improve structural stability [65,66].

At higher f-MWCNT loadings, although partial aggregation is observed in SEM analysis, the swelling degree continues to decrease. This behavior suggests that the overall network densification and interfacial bonding effects dominate over the potential swelling-promoting influence of localized aggregates. The aggregated nanotubes can still function as physical crosslinking domains, reinforcing the polymer network and further restricting chain mobility. In addition, these clusters contribute to the formation of more tortuous pathways for water diffusion rather than creating continuous channels for water penetration. Consequently, the accessibility of hydrophilic groups to water molecules remains limited, and the barrier effect of nanotubes persists despite partial agglomeration.

The water solubility of PVA/CS composite films also decreased with increasing f-MWCNT content, as summarized in Table 1. The neat PVA/CS film exhibited complete solubility (100%), which is consistent with the hydrophilic nature of both polymers and the presence of abundant hydroxyl and amino groups that facilitate strong interactions with water molecules [67]. Upon addition of f-MWCNTs, a gradual reduction in solubility was observed, reaching 68.47% at 1 wt.% filler loading. This behavior is mainly attributed to the formation of strong intermolecular interactions between functionalized nanotubes and polymer chains, resulting in improved interfacial adhesion and the development of a denser three-dimensional network structure. The presence of well-dispersed nanotubes further restricts polymer chain mobility and reduces the accessibility of hydrophilic functional groups to water molecules. In addition, nanotubes act as physical barriers that hinder the diffusion of water into the matrix, thereby suppressing the dissolution of polymer chains [68].

Overall, increasing f-MWCNT content effectively reduces both swelling degree and water solubility of PVA/CS composite films. These results indicate enhanced intermolecular bonding, reduced hydrophilicity, and improved resistance to aqueous degradation. The incorporation of f-MWCNTs therefore contributes to improved environmental stability of the composite films, making them more suitable for applications requiring controlled moisture sensitivity and structural integrity in aqueous conditions.

## 4. Conclusions

The results confirm that acid-functionalized MWCNTs significantly influence the structural organization and multifunctional performance of PVA/CS nanocomposites. The FTIR findings demonstrated the presence of strong intermolecular interactions between the functionalized nanotubes and polymer chains, which were further supported by the XRD results, indicating that crystallinity is influenced by a balance between nucleation effects and restricted molecular mobility, leading to non-monotonic structural evolution. Similar reductions in crystallinity have been widely reported in CNT-reinforced hydrophilic polymer systems, where strong interfacial interactions disrupt regular chain packing.

According to SEM analysis, functionalization improved nanotube dispersion within the polymer matrix, particularly at low filler concentrations. Uniform dispersion enhances the stress transfer efficiency and facilitates the formation of localized transport pathways, explaining the observed improvements in tensile strength, dielectric constant, and thermal conductivity.

The increase in dielectric constant is consistent with the Maxwell–Wagner–Sillars polarization mechanism, where conductive nanofillers embedded in an insulating matrix create interfacial polarization sites. Functionalization improves dispersion and interfacial compatibility, resulting in enhanced polarization behavior without premature formation of conductive networks that could cause dielectric losses. The high intrinsic thermal conductivity of CNTs and the formation of phonon transport pathways are attributed to the improved thermal conductivity. The gradual increase suggests good nanotube dispersion because severe agglomeration typically limits thermal transport efficiency. The mechanical results indicate that low nanotube concentrations improve tensile strength due to efficient stress transfer, whereas higher nanotube aggregation loadings reduce performance. This trend has been consistently reported in CNT-based polymer nanocomposites, where optimal filler loading is necessary to maximize reinforcement efficiency.

Reduced swelling and solubility indicate improved structural compactness and reduced hydrophilicity due to strong nanotube–polymer interactions. Nanotubes act as physical barriers that limit water diffusion and improve environmental stability.

In addition to the improved functional properties, the environmental implications of the developed nanocomposite films are noteworthy. The PVA/CS matrix is inherently biodegradable due to the natural origin of chitosan and the hydrolyzable structure of PVA. Although the incorporation of f-MWCNTs may slightly reduce the degradation rate by increasing structural compactness and limiting water uptake, the low filler content (≤1 wt.%) ensures that the biodegradable nature of the polymer matrix remains predominant. Consequently, the developed nanocomposite films can be considered environmentally favorable materials with controlled degradation behavior. This balance between enhanced performance and retained biodegradability makes them promising candidates for eco-friendly applications such as sustainable packaging and disposable functional materials.

From an application perspective, the simultaneous improvement in mechanical strength, thermal transport, dielectric performance, and moisture resistance significantly broadens the commercial potential of these nanocomposite films. The enhanced durability and reduced water sensitivity make them suitable for advanced packaging systems, while their improved dielectric and thermal properties enable potential use in flexible electronics, sensors, and insulating layers. The ability to achieve these multifunctional enhancements at low nanofiller concentrations further supports cost-effective and scalable production, strengthening their feasibility for real-world industrial applications.

Taken together, these findings highlight that acid-functionalized MWCNTs provide an effective strategy for simultaneously improving the multifunctional performance and environmental compatibility of PVA/CS nanocomposite films. Future studies should focus on optimizing functionalization chemistry, exploring hybrid nanofillers, and investigating electrical conductivity and gas barrier performance for advanced packaging and flexible electronic applications.

## Figures and Tables

**Table 1:** Effect of f-MWCNT concentration on the swelling degree and water solubility of PVA/CS films.

	f-MWCNT (wt.%)	Swelling Degree (%)	Water Solubility (%)
PVA/CS	0.0	140.12	100.0
	0.2	138.21	95.41
	0.5	133.54	89.15
	0.8	126.45	80.22
	1.0	118.23	68.47

## Data Availability

The original contributions presented in this study are included in the article. Further inquiries can be directed to the corresponding author.

## Abbreviations

The following abbreviations are used in this manuscript:

ASTM	American Society for testing and materials
CS	Chitosan
CNT	Carbon nanotubes
f-MWCNTs	Acid-functionalized multi-walled carbon nanotubes
FTIR	Fourier transform infrared spectroscopy
MWCNT	Multi-walled carbon nanotubes
PVA	Polyvinyl alcohol
SEM	Scanning electron microscopy
S	Solubility percentage
SR	Swelling ratio
XRD	X-ray diffraction
